# Baseline factors associated with response to ruxolitinib: an independent study on 408 patients with myelofibrosis

**DOI:** 10.18632/oncotarget.18674

**Published:** 2017-06-27

**Authors:** Francesca Palandri, Giuseppe Alberto Palumbo, Massimiliano Bonifacio, Mario Tiribelli, Giulia Benevolo, Bruno Martino, Elisabetta Abruzzese, Mariella D’Adda, Nicola Polverelli, Micaela Bergamaschi, Alessia Tieghi, Francesco Cavazzini, Adalberto Ibatici, Monica Crugnola, Costanza Bosi, Roberto Latagliata, Ambra Di Veroli, Luigi Scaffidi, Federico de Marchi, Elisa Cerqui, Barbara Anaclerico, Giovanna De Matteis, Marco Spinsanti, Elena Sabattini, Lucia Catani, Franco Aversa, Francesco Di Raimondo, Umberto Vitolo, Roberto Massimo Lemoli, Renato Fanin, Francesco Merli, Domenico Russo, Antonio Cuneo, Maria Letizia Bacchi Reggiani, Michele Cavo, Nicola Vianelli, Massimo Breccia

**Affiliations:** ^1^ Institute of Hematology “L. and A. Seràgnoli”, Sant'Orsola-Malpighi University Hospital, Bologna, Italy; ^2^ Division of Hematology, AOU 'Policlinico-V.Emanuele', Catania, Italy; ^3^ Department of Hematology, University of Verona, Verona, Italy; ^4^ Division of Hematology and BMT, Azienda Sanitaria Universitaria Integrata di Udine, Udine, Italy; ^5^ Division of Hematology, Città della Salute e della Scienza Hospital, Torino, Italy; ^6^ Division of Hematology, Azienda Ospedaliera 'Bianchi Melacrino Morelli', Reggio Calabria, Italy; ^7^ Division of Hematology, Ospedale S. Eugenio, Roma, Italy; ^8^ Division of Hematology, ASST Spedali Civili di Brescia, Brescia, Italy; ^9^ Unit of Blood Diseases and Stem Cell Transplantation, ASST Spedali Civili di Brescia, Brescia, Italy; ^10^ Division of Hematology, IRCCS AOU San Martino-IST, Genova, Italy; ^11^ Department of Hematology, A.O. Arcispedale Santa Maria Nuova – IRCCS, Reggio Emilia, Italy; ^12^ Division of Hematology, University of Ferrara, Ferrara, Italy; ^13^ Division of Hematology and Bone Marrow Transplant, IRCCS San Martino-IST, Genova, Italy; ^14^ Division of Hematology, AOU of Parma, Parma, Italy; ^15^ Department of Hematology and Bone Marrow Transplantation, A.O. of Piacenza, Italy; ^16^ Division of Cellular Biotechnologies and Hematology, University Sapienza, Roma, Italy; ^17^ Division of Hematology, Policlinico Tor Vergata, Roma, Italy; ^18^ Division of Hematology, Ospedale S. Giovanni, Roma, Italy; ^19^ Department of Life and Reproduction Sciences, Section of Clinical Biochemistry, University of Verona, Verona, Italy; ^20^ Division of Cardiology, University of Bologna, Bologna, Italy

**Keywords:** myelofibrosis, splenomegaly, response, ruxolitinib, predictive factors

## Abstract

In patients with Myelofibrosis (MF) treated with ruxolitinib (RUX), the response is unpredictable at therapy start. We retrospectively evaluated the impact of clinical/laboratory factors on responses in 408 patients treated with RUX according to prescribing obligations in 18 Italian Hematology Centers. At 6 months, 114 out of 327 (34.9%) evaluable patients achieved a spleen response. By multivariable Cox proportional hazard regression model, pre-treatment factors negatively correlating with spleen response were: high/intermediate-2 IPSS risk (*p*=0.024), large splenomegaly (*p*=0.017), transfusion dependency (*p*=0.022), platelet count <200×10^9^/l (*p*=0.028), and a time-interval between MF diagnosis and RUX start >2 years (*p*=0.048). Also, patients treated with higher (≥10 mg BID) average RUX doses in the first 12 weeks achieved higher response rates (*p*=0.019). After adjustment for IPSS risk, patients in spleen response at 6 months showed only a trend for better survival compared to non-responders. At 6 months, symptoms response was achieved by 85.5% of 344 evaluable patients; only a higher (>20) Total Symptom Score significantly correlated with lower probability of response (*p*<0.001). Increased disease severity, a delay in RUX start and titrated doses <10 mg BID were associated with patients achievinglower response rates. An early treatment and higher RUX doses may achieve better therapeutic results.

## INTRODUCTION

Myelofibrosis (MF) is a chronic myeloproliferative neoplasm (MPN) primarily characterized by dysregulation of the JAK-STAT pathway, that is thought to be responsible for increased myeloproliferation and abnormal production of pro-inflammatory cytokines [1]. MF can present as primary disease (PMF) or post essential thrombocythemia (PET-MF) or post polycythemia vera (PPV-MF). The clinical hallmarks of both primary and secondary MF are splenomegaly, constitutional (specifically, fever, weight loss and night sweats) and/or disease-related (i.e. fatigue, pruritus and abdominal pain) symptoms, and cytopenias (mainly, anemia) [2, 3]. MF results in severely impaired quality of life and reduced survival, particularly in patients with high and intermediate-2 risk disease according to the International Prognostic Score System (IPSS) [4].

Ruxolitinib (RUX) is a potent and selective JAK1/JAK2 inhibitor that has demonstrated superiority over placebo [5, 6] and over best available therapy (BAT) in the phase 3 Controlled MyeloFibrosis Study with Oral JAK Inhibitor Treatment II (COMFORT-II) trial [7, 8]. In this latter trial, 32% of patients randomized to ruxolitinib achieved ≥35% decrease in spleen volume at week 24 and many patients had also marked reductions in myelofibrosis-associated symptoms. In the first report of the Phase 3b expanded access JAK Inhibitor RUxolitinib in Myelofibrosis Patients (JUMP) trial, 62.3% of patients achieved a ≥50% reduction from baseline in palpable spleen length at 48 weeks, and around 50% of patients had a symptoms response according to different scales [9]. While extra-hematological toxicity was mild and infrequent, grade 3-4 anemia and thrombocytopenia were observed in 33% and 12.5% of patients, respectively. Based on these efficacy and safety data, RUX has become the first and still only JAK1/2 inhibitor commercially available for the treatment of MF. To date, treatment is commonly triggered by the appearance or progression of significant clinical needs, and there are no baseline features that may predict responses and help selecting patients who are more likely to benefit from RUX therapy.

Here, we report a large cohort of MF patients treated with RUX and evaluated for response according to the International Working Group for Myeloproliferative Neoplasms Research and Treatment (IWG-MRT) and European LeukemiaNet (ELN) criteria [10], with the aims to: 1) provide independent data on type and rate of homogeneously defined responses; 2) evaluate pre-treatment clinical/laboratory factors associated with responses; 3) investigate the role of RUX doses on efficacy measures; 4) explore the potential association of spleen response with survival.

## RESULTS

### Study cohort

Between June 2011 and Apr 2016, 408 patients with PMF (n. 222, 54.4%), PET-MF (n. 113, 27.7%) or PPV-MF (n. 73, 17.9%) were treated with RUX in 18 Italian Hematology Centers and were included in the study. Overall, 160 patients received RUX as per compassionate or commercial use, while 248 (60.8%) patients were first enrolled in the JUMP trial [9], which was closed for enrolment in September 2014. From January 2015 onwards, all patients received the drug outside clinical trials. The total observation time was 903.5 patient-years; the time spent in the JUMP trial accounted for 363.2 patient-years. Patients received a re-evaluation of hematology parameters, marrow histology, fibrosis grading and karyotype before the start of RUX. Table 1 summarizes main baseline clinical and laboratory data of the entire cohort. Patients treated off-study had similar baseline features as compared to patients first enrolled in the JUMP trial. However, they were more frequently at intermediate2/high IPSS risk (88.8% vs 81.5%, *p*=0.048).

**Table 1:** Patients’ characteristics at ruxolitinib start

Characteristics	Patients (n. 408)
**Male sex, no (%)**	230 (56.4%)
**Median age, years (range)**	68.5 (26.5 – 89.0)
**Primary Myelofibrosis, no (%)**	222 (54.4%)
** Age >65 years, no (%)**	259 (63.5%)
**IPSS intermediate-2/high, no (%)**	344 (84.3%)
**Median hemoglobin, g/dl (range)**	10.7 (7 – 16.7)
** Hemoglobin <10 g/dl**	173 (42.4%)
**Transfusion dependence, no (%)**	114 (27.9%)
**Median platelet, ×10**^9^**/l (range)**	256.5 (50 – 1632)
** Platelet >200 ×10**^9^**/l**	259 (63.5%)
** Platelet <100 ×10**^9^**/l**	39 (9.6%)
**Constitutional symptoms, no (%)**	220 (53.9%)
**Palpable spleen, no (%)**	394 (96.6%)
** Spleen ≥10 cm, no (%)**	262 (64.2%)
***JAK2*^V617F^ mutation, no (% on 347 evaluable)**	281 (81.0%)
**Unfavorable karyotype, no (% on 212 evaluable)**	17 (8.0%)
**Grade 3 marrow fibrosis, no (% on 378 evaluable)**	108 (28.6%)
**Time from MF diagnosis to RUX start >2 years**	185 (45.3%)
**Mean time from MF diagnosis to RUX start, months (SD)**	44.4 (58)
**RUX starting dose**	
** 5 mg BID**	49 (12.0%)
** 10 mg BID**	30 (7.4%)
** 15 mg BID**	108 (26.5%)
** 20 mg BID**	221 (54.2%)

Karyotype was abnormal in 55 (25.9%) out of 212 evaluable patients. In 17 cases (8%) an unfavorable karyotype was detected, specifically: trisomy 8 (5 patients), complex (5 patients), del7 (3 patients), del5 (3 patients), and trisomy 1 (1 patient).

Marrow fibrosis was evaluable in 378 (92.6%) patients and was grade 1 in 107 (28.3%), grade 2 in 163 (43.1%), and grade 3 in 108 patients. Full molecular data were available for 323 patients (79.2%): *JAK2*^V617F^ was present in 87%, *CALR* mutations in 8%, and *MPL*^W515K/L^ in 1%; 4% of the patients were triple negatives. Twenty-four patients (5.9%) were *JAK2*^V617F^-negative but did not receive further molecular evaluation; in 61 (14.9%) cases, no molecular data was available. Most (57.9%) *JAK2*^V617F^-positive patients were homozygous; median allele burden was 80% (range, 2%–99%). Median follow-up from MF diagnosis was 3.8 years (range, 0.3-29.2) and median RUX exposure was 20 months (range, 3-56.2).

### Ruxolitinib doses and causes of discontinuations

**Figure 1:**
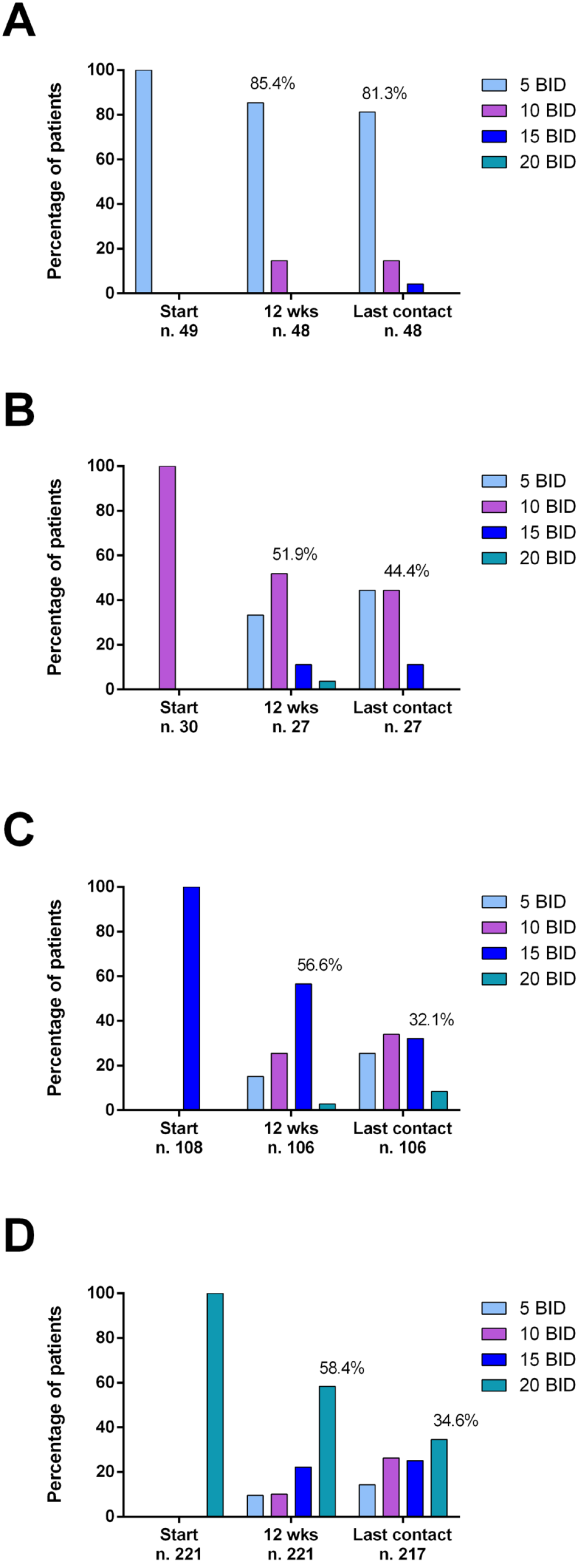
Proportion of patients treated with different doses of ruxolitinib over time, after stratification according to ruxolitinib starting doses (**A**: 5 mg BID; **B**: 10 mg BID; **C**: 15 mg BID; **D**: 20 mg BID). Percentages are calculated on evaluable patients at each time point. Ruxolitinib starting doses were mainly administered according to prescribing information (i.e.: 5 mg BID if platelet between 50 and 99 ×10^9^/l, 15 mg BID if platelet between 100 and 199 ×10^9^/l, 20 mg BID if platelet ≥ 200 ×10^9^/l).

RUX starting dose was 20 mg BID, 15 mg BID, 10 mg BID and 5 mg BID in 221 (54.2%), 108 (26.5%), 30 (7.3%), and 49 (12%) patients, respectively. Many (39.3%) patients had a dose modification during the first 12 weeks of therapy (dose decrease in 91.1% of the cases). Particularly, 40.6% and 41.7% of patients that started ruxolitinib at the dose of 15 mg and 20 mg BID, respectively, underwent a dose reduction at 12 weeks. Conversely, 14.6% and 14.8% of patients that initiated ruxolitinib at the dose of 5 and 10 mg BID, respectively, were eventually able to increase ruxolitinib dose (Figure 1). The average daily dose was 28.3 mg for patients starting at 20 mg BID, 22.9 mg for patients starting at 15 mg BID, 17.2 mg for patients starting at 10 mg BID, and 11.7 mg for patients starting at 5 mg BID.

Overall, 146 (35.8%) patients discontinued RUX after a median time of 13 months. More specifically, 32 out of 379 evaluable patients discontinued RUX within the first 6 months of therapy, 68 out of 334 evaluable patients within 12 months and 93 out of 308 evaluable patients within 18 months, for a discontinuation rate of 8.4%, 20.4% and 30.2% at 6, 12 and 18 months, respectively. Reasons for RUX discontinuations were: lack of response (27 patients, 18.5%); loss of response (15, 10.3%); drug-related toxicity (27.4%, specifically: 24 patients for thrombocytopenia, 16.4%; 9 for severe infections, 6.2%; 7 for anemia, 4.8%); disease progression with/without acute evolution (13 patients, 8.9%); death (20, 13.7%); allogeneic transplant (13, 8.9%); second neoplasia (6, 4.1%); other causes (12 patients, 8.2%).

A total of 30 (7.4%) patients developed acute leukemia, after a median time from RUX start of 13.1 months; in 7 cases, RUX was suspended before the diagnosis of AL. The incidence rate of acute leukemia was 1.3 per 100 patient-years from MF diagnosis and 3 per 100 patient-years from RUX start.

Ninety-six (23.6%) patients died after a median time from RUX start of 15.4 months (range 1.5-56.7). Causes of death were, specifically: progression of myelofibrosis (37 patients, 38.5%), evolution into AL (16, 16.7%), infections (13, 13.5%), heart disease (9, 9.4%), thrombotic/hemorrhagic events (8, 8.3%), allogeneic transplantation (4, 4.2%), second neoplasia (2, 2.1%), and other causes (7, 7.3%). Overall survival at 2 years from RUX start was 78.5%. Survival was not influenced by the type of MF diagnosis (primary versus secondary MF) (log-rank *p*=0.53). As expected, OS at 2 years was significantly influenced by dynamic-IPSS (DIPSS) score [11] at RUX start (94.6%, 82.2% and 51.9% in intermediate-1, intermediate-2 and high risk patients, respectively, *p*<0.001).

### Spleen response and baseline factors correlating with response

**Figure 2:**
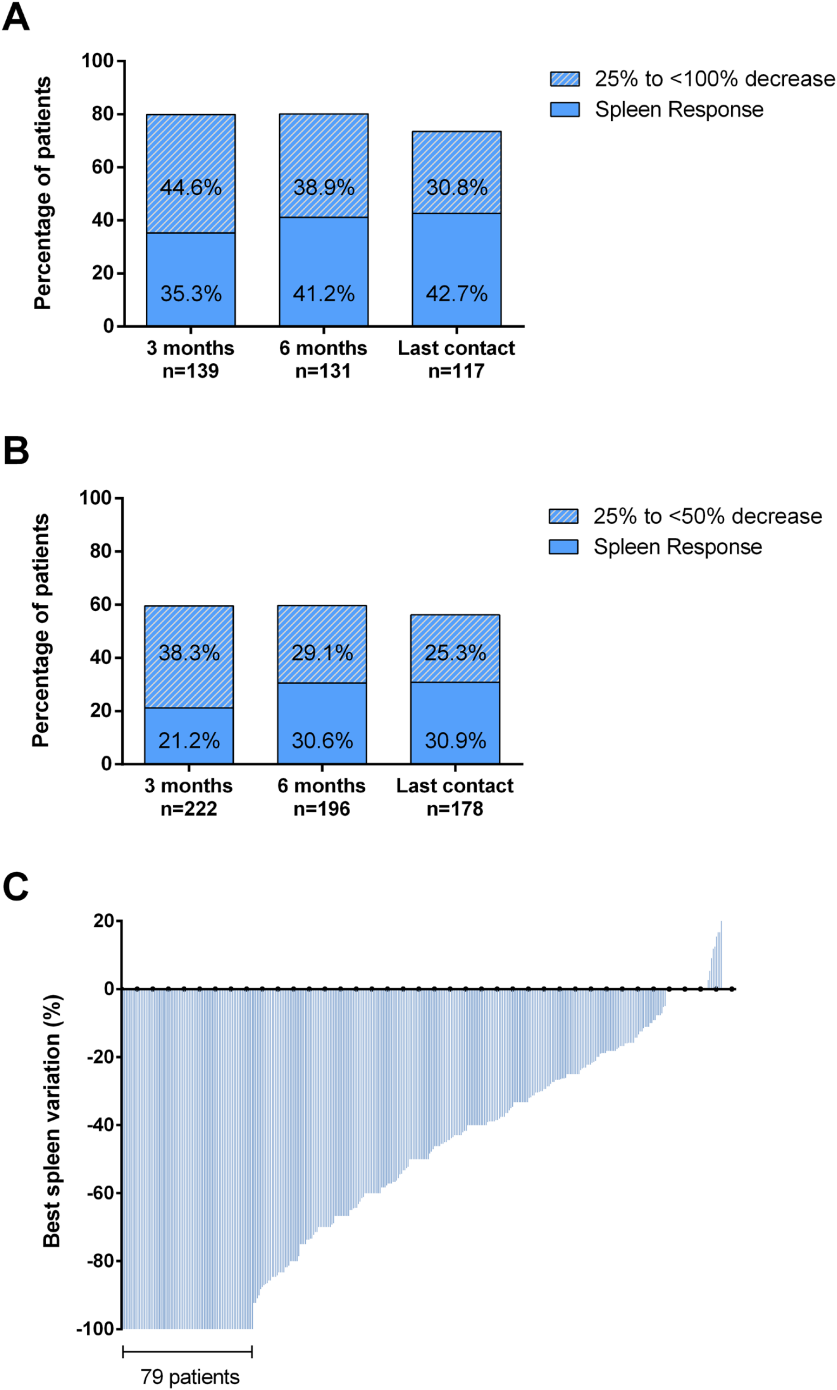
Spleen response A baseline splenomegaly palpable at <5 cm was not eligible for spleen response. **(A)** Evaluable patients with a baseline spleen palpable between 5 and 10 cm below left costal margin. Spleen response: 100% decrease (not palpable spleen). **(B)** Evaluable patients with a baseline spleen palpable >10 cm below left costal margin. Spleen response: ≥50% decrease in palpable spleen length. **(C)** Best percent change from baseline in palpable spleen length at any time. Each bar represents data from an individual patient.

**Figure 3:**
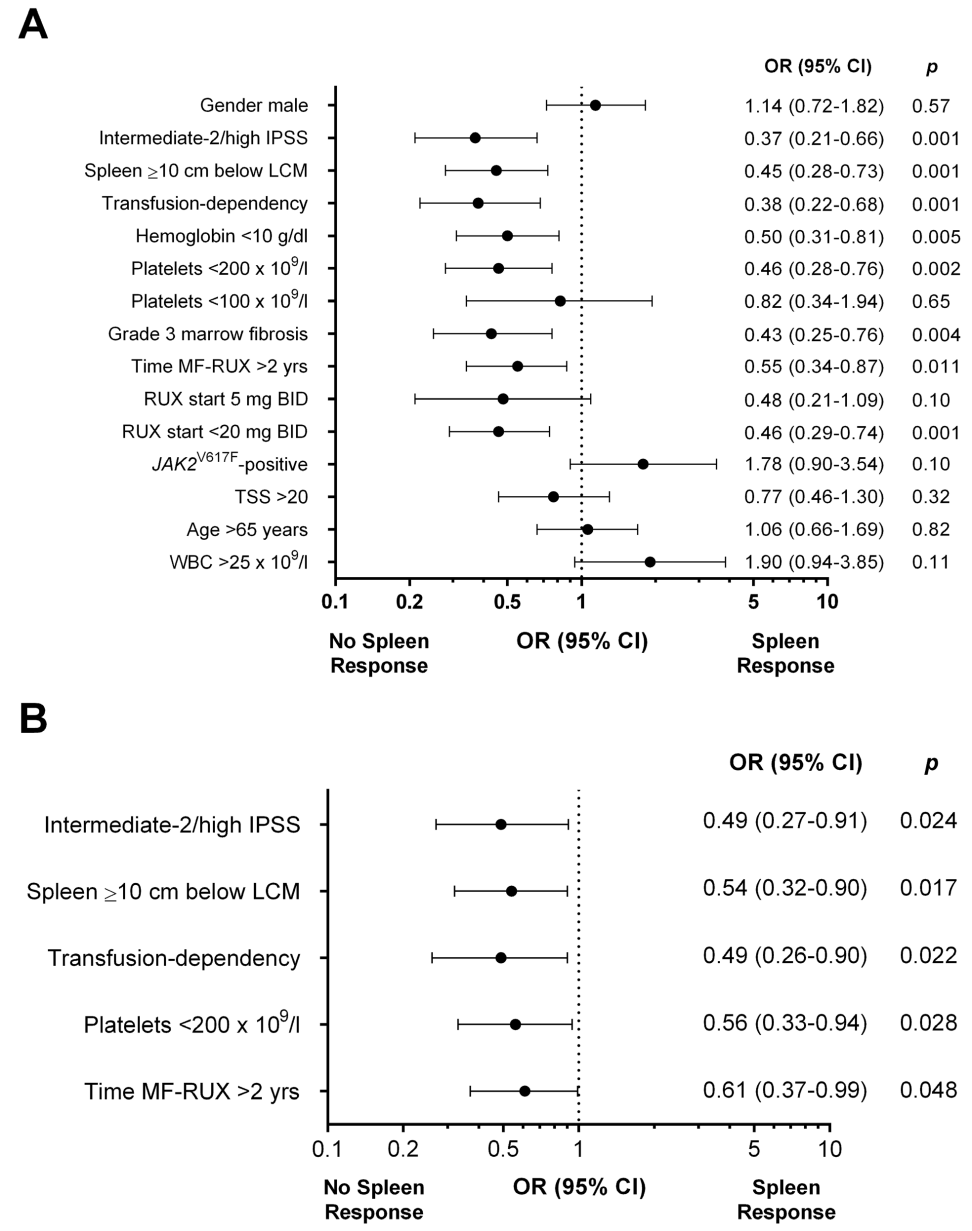
Univariate **(A)** and multivariable **(B)** logistic regression models of baseline factors predictive for spleen response at 6 months in patients treated with ruxolitinib. The area under the ROC curve was 0.69 and the H-L test reported a *p* value of 0.79. IPSS: International Prognostic Score System. TSS: Total Symptom Score. Fibrosis was evaluated according to the European Consensus Grading System [33].

Spleen response was evaluable in 361 out of 408 (88.5%) patients. A baseline splenomegaly palpable at <5 cm was not eligible for spleen response [9]. A total of 152 (42%) patients achieved a spleen response at any time by 3 years from RUX start. The overall rate of spleen responses was comparable in patients enrolled in the JUMP trial (45.6%) or treated in a “real-life” setting (36.7%) (*p*=0.09). At 3 and 6 months, the response was achieved by 26.6% and 34.9% of 361 and 327 evaluable patients, respectively. The rate of spleen reduction at least ≥25% from baseline was significantly higher in patients with spleen palpable between 5 and 10 cm below LCM respect to patients with spleen >10 cm (79.9% vs 59.5%, *p*<0.001 at 3 months and 80.1% vs 59.7%, *p*<0.001 at 6 months) (Figure 2A and 2B). In 79 (21.9%) cases, spleen became not palpable (Figure 2C). Figure 3 reports the correlations between main baseline clinical and laboratory features and subsequent spleen response at 6 months. In univariate analysis, pre-treatment factors negatively correlating with spleen response were: IPSS risk intermediate-2/high (*p*=0.001), spleen palpable ≥10 cm below LCM (*p*=0.001), transfusion-dependency (*p*=0.001), time interval between MF diagnosis and RUX start >2 years (*p*=0.011), anemia (Hb <10 g/dl, *p*=0.005), grade 3 marrow fibrosis (*p*=0.004), platelet count <200×10^9^/l (*p*=0.002), and RUX starting dose <20 mg BID (*p*=0.001) (Figure 3A). The diagnosis of PMF versus PET/PPV-MF was not significantly associated with spleen response, that was achieved by 25.7% and 26.9% of the patients, respectively (*p*=0.83). In multivariable regression logistic analysis, 5 variables remained significantly associated with a lower probability of spleen response: high/intermediate-2 IPSS risk (*p*=0.024); a large (≥10 cm below LCM) splenomegaly (*p*=0.017), transfusion dependency (*p*=0.022), platelet count <200×10^9^/l (*p*=0.028), and a time-interval between MF diagnosis and RUX start >2 years (*p*=0.048) (Figure 3B). We also evaluated the additional prognostic value of the “number” of predictive factors presented by each individual patient. Specifically, 64 (15.7%) patients presented one factor, 123 (30.1%) presented two factors, while 209 (52.1%) presented three or more baseline features among the five associated with worse response. The presence of three or more factors was significantly associated with lower probability of spleen response at 6 months compared to patients carrying ≤2 factors (26.7% vs 49.6%, respectively; *p*<0.001).

Notably, a platelet count <200×10^9^/l corresponded to a RUX starting dose lower than 20 mg BID. Accordingly, the rate of spleen response at 6 months was significantly higher in patients who started RUX at 20 mg BID (42.7% versus 26.8% in patients starting RUX with 10 or 15 mg BID, *p*=0.008, and 42.7% versus 21.6% in patients starting with 5 mg BID, *p*=0.017) (Supplementary Figure 1A). Also, patients titrated at average doses ≥10 mg BID during the first 12 weeks of therapy achieved more frequently a spleen response at 6 months compared to patients that received lower average doses (Supplementary Figure 1B).

**Figure 4:**
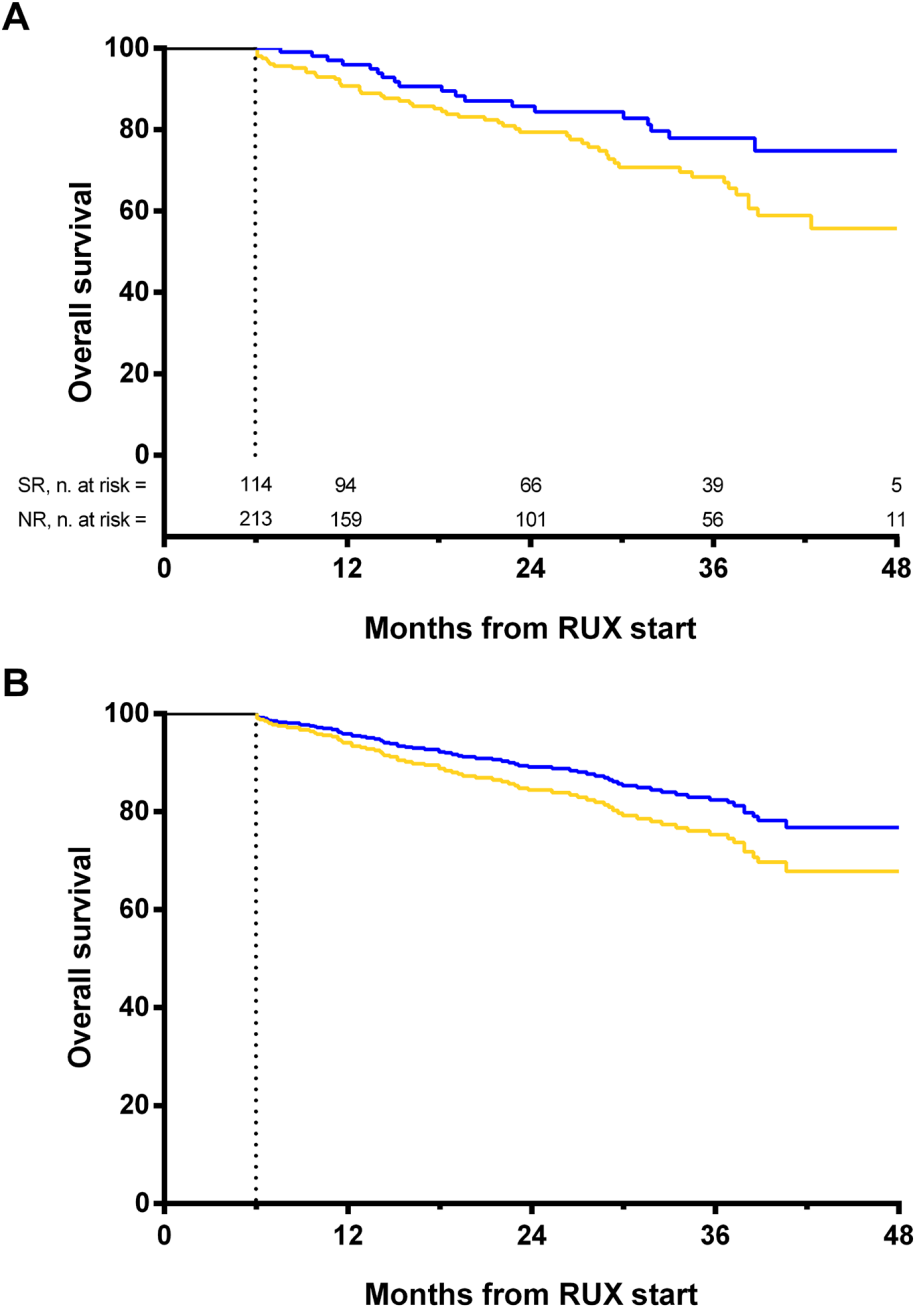
Landmark analyses by spleen response at 6 months A 6-month time after the initiation of therapy was selected as a landmark for conducting the analysis of survival by response. Only patients alive at 6 months were included in the analysis, separated into two response categories according to whether they have had a spleen response at that time-point. **(A)** Unadjusted survival rate calculated with Kaplan-Meier. Survival probability at 3 years from ruxolitinib start was 77.9% in patients achieving a spleen response at 6 months (blue line, n=114) and 68.4% in patients without a spleen response (yellow line, n=213) (Log-rank, *p*=0.034). **(B)** Overall survival estimation adjusted for IPSS score (HR: 1.47, 95% CI: 0.87–2.49, *p*=0.151). The dashed line on the x-axis represents the 6-months landmark point. SR: spleen response. NR: no response.

By landmark analysis, overall survival was significantly better in patients achieving a spleen response at 6 months (Figure 4A). However, when the overall survival estimation was adjusted for the IPSS score (intermediate-1 vs intermediate-2/high) only a trend for statistical significance was observed (HR: 1.47, 95% CI: 0.87–2.49, *p*=0.151) (Figure 4B).

Notably, the present analysis was confirmed when using the DIPSS instead of IPSS score. In multivariable analysis, intermediate 2/high DIPSS risk score remained negatively associated with the probability of achieving a spleen response at 6 months (OR=0.50, 95% CI 0.27-0.94, *p*=0.032). By adjusting the survival curve for DIPSS, only the trend for statistical significance was observed (*p*=0.131).

### Symptoms response and baseline factors correlating with response

Overall, 359 (89.3%) out of 402 evaluable patients achieved a symptoms response by 3 years from therapy start (Supplementary Figure 2A). The overall rate of symptoms responses were comparable in patients enrolled in the JUMP trial (89.8%) or treated off study (87.9%) (*p*=0.54). At 3 and 6 months, 315 out of 402 (78.4%) and 294 out of 344 (85.5%) evaluable patients achieved a symptoms response. Notably, no significant correlation was found between RUX starting and 12-weeks titrated doses and the rate of symptoms responses at 6 months (Supplement Figure 2B and 2C, respectively).

**Figure 5:**
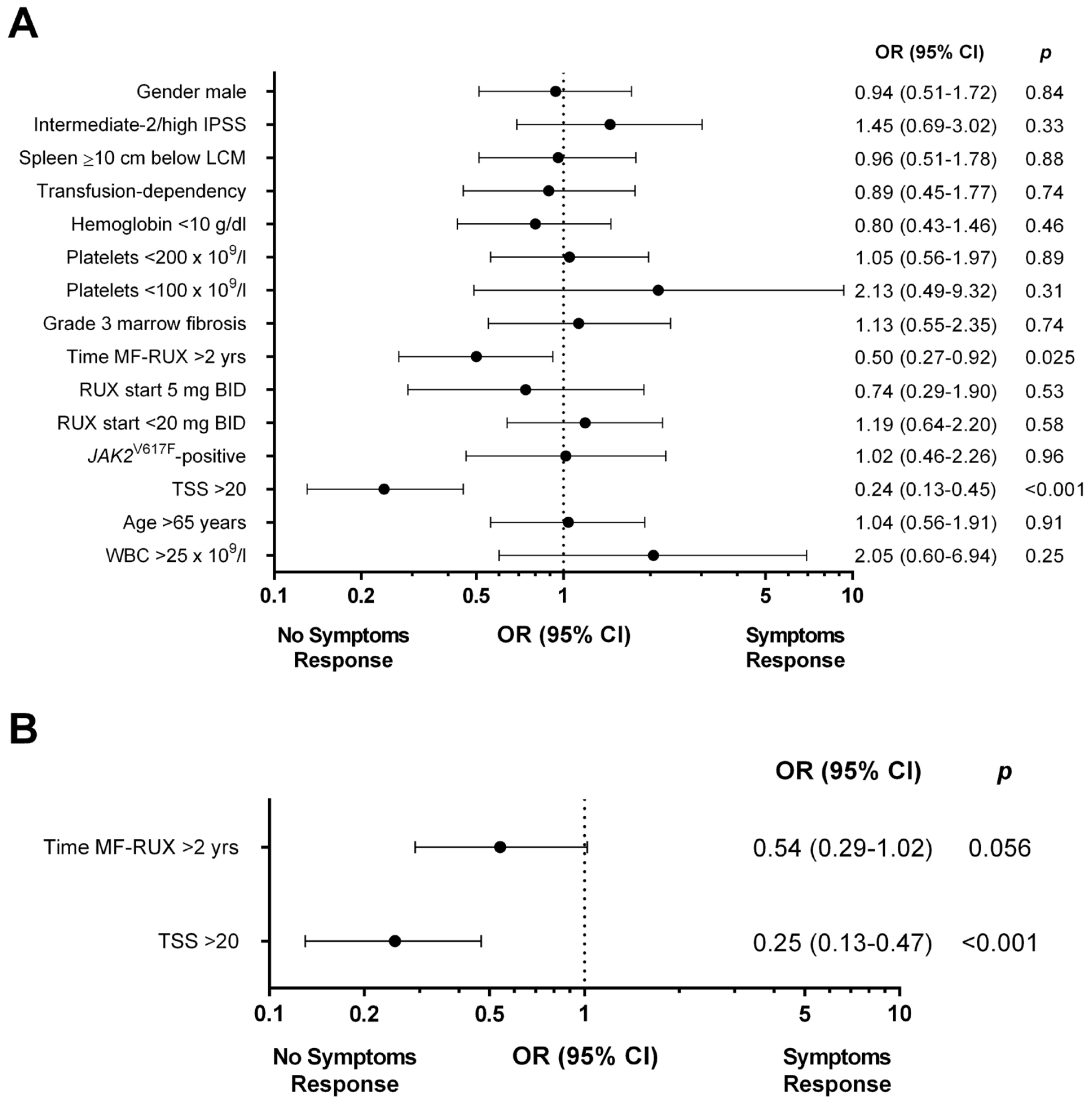
Univariate **(A)** and multivariable **(B)** logistic regression models of baseline factors predictive for symptoms response at 6 months in patients treated with ruxolitinib. The area under the ROC curve was 0.70 and the H-L test reported a *p* value of 0.47. IPSS: International Prognostic Score System. TSS: Total Symptom Score.

In univariate analysis, patients with a Total Symptom Score (TSS) >20 and with a time-interval between MF diagnosis and RUX start longer than 2 years had a significantly lower probability of achieving a symptoms response at 6 months (70.1% vs 90.7% of patients with lower TSS, *p*<0.001; and 80.5% vs 89.2% of patients with time-interval <2 years, *p*=0.025, respectively) (Figure 5A).

The diagnosis of PMF versus PET/PPV-MF was not significantly associated with symptoms response, that was achieved by 85.9% and 85% of the patients, respectively (*p*=0.88). In multivariable analysis, a baseline TSS >20 (OR: 0.25, 95% CI: 0.13–0.47; *p*<0.001) remained significant; a delay in treatment start showed only a trend for statistical significance (OR: 0.54, 95% CI: 0.29–1.02, *p*=0.056) (Figure 5B).

## DISCUSSION

Ruxolitinib has significantly improved the therapeutic scenario of MF patients with splenomegaly and systemic symptoms. Nonetheless, approximately 50% of patients do not achieve a satisfactory spleen and/or symptom response, and no predictors of response have yet been identified to select patients who could benefit most from therapy.

The present study includes a large cohort of MF patients who received RUX for a considerably long period of time and were homogeneously evaluated according to the 2013 IWG-MRT criteria. In previous studies, using different response criteria, spleen response at 24 weeks varied significantly (COMFORT-1: 41.9%; COMFORT-2: 32%; JUMP: 56.9%; and ROBUST 54.2%) [5, 7, 9, 12]. We observed a rate of spleen responses of 34.9% that was comparable to that (32%) observed in a previous retrospective study using the IWG-MRT criteria [13]. Conversely, our rate of symptoms responses was significantly higher than in previous studies (85.5% versus around 40-50% in COMFORT-1/COMFORT-2/JUMP trials and 54.2% in the ROBUST study) [12]. Notably, our study included subjects that were treated at different hematology centers, some enrolled on an industry-sponsored study, others not. Nonetheless, spleen and symptom response rates were comparable in the two cohorts (Supplementary Table 1).

The first result of this study is that spleen response was inversely associated with MF severity, in terms of large splenomegaly, high/intermediate-2 IPSS risk, transfusion dependency and lower platelet count. Similarly, the probability to achieve a symptoms response was significantly lower in patients with higher burden of the disease (specifically, patients with baseline TSS >20). The relation between a more advanced disease and inferior responses may be expected, since patients with end-stage diseases are commonly more resistant to treatments. Indeed, the positive association between intermediate-1 IPSS risk and spleen responses supports the results of the UK ROBUST study as well as those from real-world clinical evidence of ruxolitinib use in patients with lower risk MF [12, 14, 15]. It is acknowledged that, in absence of effective therapy, splenomegaly and systemic symptoms progressively worsen over time [4, 16]. Accordingly, in our study a time interval between MF diagnosis and ruxolitinib start longer than 2 years was significantly associated with decreased probability of spleen response. These data support the rationale for ongoing clinical studies evaluating if early treatment may achieve better therapeutic results.

Patients achieving a spleen response at 6 months had better survival compared to non-responders, although the difference did not maintain statistical significance after adjustment for the IPSS risk. Accordingly, a phase I/II study from MD Anderson Cancer Center and a recent pooled analysis of the COMFORT studies showed that spleen response was associated with better survival in RUX-treated patients, while a 5 dl increase from baseline in spleen size correlated with worse outcome [17, 18]. In this latter study, a positive correlation of greater spleen size reduction with a reduced risk of death was not observed in patients in the combined control group. Overall, further data are needed to clarify whether spleen reduction at 6 months might be considered as a surrogate marker for survival specific for RUX-treated patients.

The second result of this analysis is that RUX starting and titrated dose may influence spleen, but not symptoms, response. A trend for higher response rates in patients receiving titrated doses ≥10 mg BID was first highlighted in the ruxolitinib Phase 1-2 trial, and current expert recommendation suggest to maintain the maximum tolerated dose [19–22]. Additionally, ruxolitinib dose intensity, expressed as median cumulative dose, was found to be independently associated with spleen responses, together with higher *JAK2*^V617F^ allele burden, in a recent study on 69 patients [13]. Here, RUX starting dose has been shown to significantly correlate with better responses irrespective of subsequent dose modifications. Nonetheless, patients that received stable doses of at least 10 mg BID during the first 12 weeks of therapy had better spleen response rates at 6 months. Particularly, patients titrated at 20 mg BID achieved the best response rates (42.3%). Overall, these data reinforces the recommendation to start with the higher possible dose according to baseline platelet count, and to maintain the maximum tolerated, at least during the first 3 months of treatment.

One of the limitations to our study is that, in absence of prospectively validated criteria, we adopted the 2013 IWG-MRT criteria to assess responses. We acknowledge that these criteria are only a consensus statement, and may not be widely accepted. Second, marrow biopsies were not centrally reviewed and molecular analyses including driver and subclonal mutations [23–26] were not routinely and homogeneously performed in all Centers. The absence of extensive molecular evaluations, together with a preferential use of RUX in *JAK2*^V617F^-mutated patients, may also explain the non-usual distribution of driver mutations. Thus, the present study may not properly address the role of these pivotal biological data on responses to therapy. However, a higher degree in marrow fibrosis was correlated, in univariate analysis, with lower spleen response, supporting the known negative role of marrow fibrosis on MF prognosis [27–30].

In conclusion, a more advanced disease, a delay in ruxolitinib start and lower ruxolitinib doses identified patients with lower response rates. Taken together, these data may suggest the use of ruxolitinib at an early stage of the disease, when splenomegaly and/or systemic symptoms are milder and likely to be more responsive, possibly leading to a survival advantage.

## MATERIALS AND METHODS

### Patients and treatment

A multicenter observational study on WHO-defined MF was conducted in 18 Italian Hematology Centers. Subjects were enrolled into the JAK Inhibitor rUxolitinib in Myelofibrosis Patients (JUMP) trial (ClinicalTrials.gov Identifiers: NCT01493414) or treated off-study. Data were extracted from an electronic database that included consecutive patients treated with RUX from June 2011. Data cut-off was July 2016.

All treatments for MF, as well as baseline clinical/laboratory features and outcome measures (including evolution into acute leukemia, death and spleen/symptoms responses) were recorded. Diagnosis of PMF and PET/PPV-MF was made according to the WHO 2008 [31] or the IWG-MRT criteria [32], respectively. Histological examination was performed at local institutions; marrow fibrosis was graded according to the European Consensus Grading System [33]. Diagnosis of acute leukemia (AL) was made according to WHO criteria [34].

Spleen/symptoms responses and transfusion dependency were assessed at 3 and 6 months after treatement start and at last contact during ruxolitinib therapy. All responses were defined according to 2013 IWG-MRT/ELN criteria [10]. Specifically, a spleen response was defined as disappearance of splenomegaly in patients with baseline splenomegaly palpable at 5-10 cm below the LCM or as a decrease by ≥50% by palpation in case of a baseline splenomegaly palpable at >10 cm. A baseline splenomegaly that is palpable at <5 cm was not eligible for spleen response. Loss of response was defined as any increase in spleen size not meeting the initial response criteria. Due to its retrospective and observational nature, this study includes only evaluations performed in routine care; therefore, spleen responses did not receive confirmation by imaging techniques.

A symptoms response required a ≥50% reduction in the Myeloproliferative Neoplasm Symptom Assessment Form Total Symptom Score (MPN-SAF TSS) [35]. The MPN-SAF TSS was assessed by the patients themselves and this includes fatigue, concentration, early satiety, inactivity, night sweats, itching, bone pain, abdominal discomfort, weight loss, and fevers. Scoring is from 0 (absent/as good as it can be) to 10 (worst imaginable/as bad as it can be) for each item. The MPN-SAF TSS is the summation of all the individual scores (0-100 scale) [35]. Transfusion dependency was also defined according to 2013 IWG-MRT criteria, as transfusions of at least 6 units of RBC in the 12 weeks prior to ruxolitinib start, in the absence of bleeding or treatment-induced anemia.

The study was approved by the Institutional Review Board of each Institution and was conducted according to the Helsinki declaration.

### Molecular and cytogenetic analysis

*JAK2*^V617F^ allele-burden was assessed in granulocyte DNA by quantitative polymerase chain reaction (PCR)–based allelic discrimination assay (ipsogen JAK2 MutaQuant Kit) on 7900 HT Fast Real-Time-PCR System (Applied Biosystems) or by semi-quantitative PCR [36]. *CALR* exon 9 and *MPL* mutations were investigated as described elsewhere [37]. Chromosome banding analysis was performed on marrow cells by standard banding techniques according to the International System for Human Cytogenetic Nomenclature [38].

### Statistical analysis

Continuous variables were expressed as median and ranges and categorical variables were presented as frequencies and percentages. Comparisons between groups were performed with Chi-square test and Two-sample Wilcoxon rank-sum (Mann-Whitney) test for categorical and continuous variables, respectively. Multivariable logistic regression analyses were conducted on variables with *p*<0.1 at univariate analysis.

Collinearity (multicollinearity) occurs when there are high correlations among predictor variables, leading to unreliable and unstable estimates of regression coefficients. To avoid this problem a common solution is to remove highly correlated predictors from the model, because they supply redundant information. Collinearity amongst variables was detected by means of Pearson correlation test and grade 3 fibrosis, hemoglobin <10 g/dl, and RUX start dose <20 mg BID variables were found to be associated with the other factors; hence, they were excluded from the analysis.

Models building followed a backward-stepwise approach. For the multivariable logistic regression the model discrimination (area under the Receiver Operator Characteristic [ROC] curve) and calibration (Hosmer-Lemeshow [H-L] test) were reported. Survival analysis was performed by means of Kaplan-Meier (KM) product-limit estimate and differences between KM estimates were evaluated using the Log-rank test. To assess spleen response role as independent predictor of survival a multivariable Cox proportional hazard regression model was fitted to the data adjusting for the IPSS score (intermediate-1 vs intermediate-2/high). All tests were 2-sided and a *p* value less than 0.05 was considered statistically significant. Analyses were performed with IBM SPSS Statistics 22 (IBM Analytics) and GraphPad Prism 6 (GraphPad Software).

## SUPPLEMENTARY MATERIALS FIGURES AND TABLE


