# Supplementary material for: Baseline factors associated with response to ruxolitinib: an independent study on 408 patients with myelofibrosis
Source: Oncotarget. 2017 Jun 27;8(45):79073–86. doi: 10.18632/oncotarget.18674 (PMC5668021; doi:10.18632/oncotarget.18674)
Supplement: Supplementary file 1 [file oncotarget-08-79073-s001.pdf]

Baseline factors associated with response to ruxolitinib: an independent study on 408 patients with myelofibrosis

SUPPLEMENTARY MATERIALS

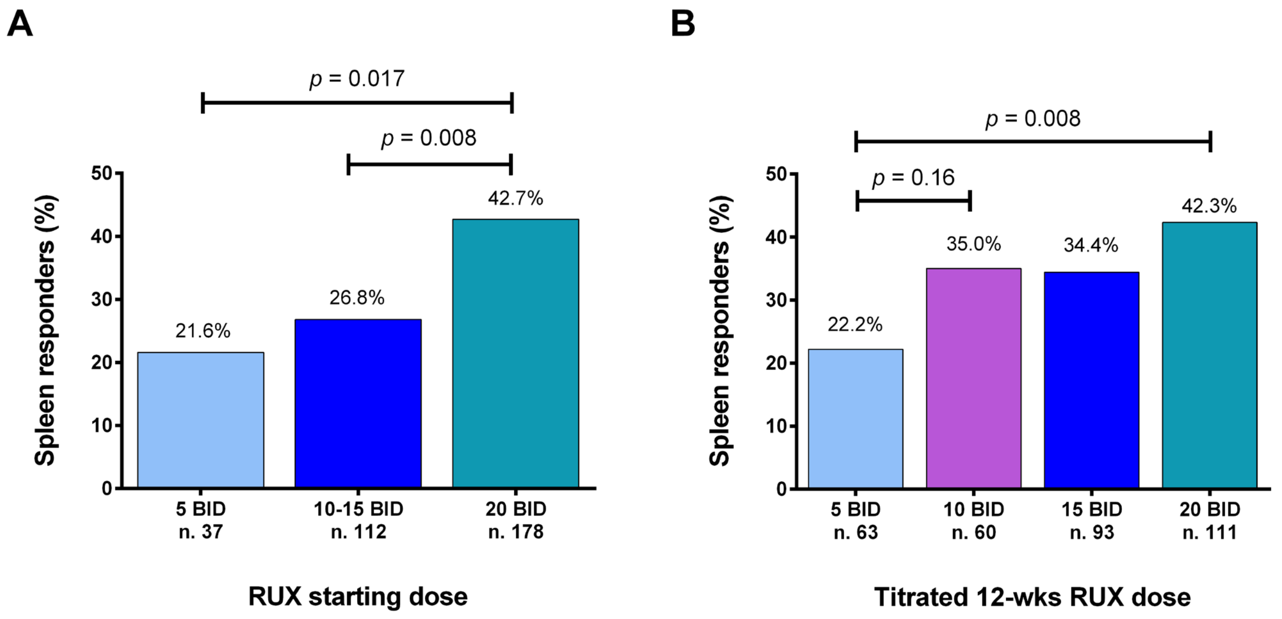

Supplementary Figure 1: Spleen response according to ruxolitinib starting **(A)** and 12-weeks titrated **(B)** dose. Percentages are calculated on 327 evaluable patients. Patients starting with doses higher or equal to 20 mg BID had the greatest probability of spleen response (42.7% versus 25.5% of patients starting with lower doses, *p*=0.001); also, 100 patients out of 264 (37.9%) maintaining average doses ≥10 mg BID during the first 12 weeks of therapy had a spleen response at 6 months versus 14 out of 63 (22.2%) patients titrated at lower doses (*p*=0.019).

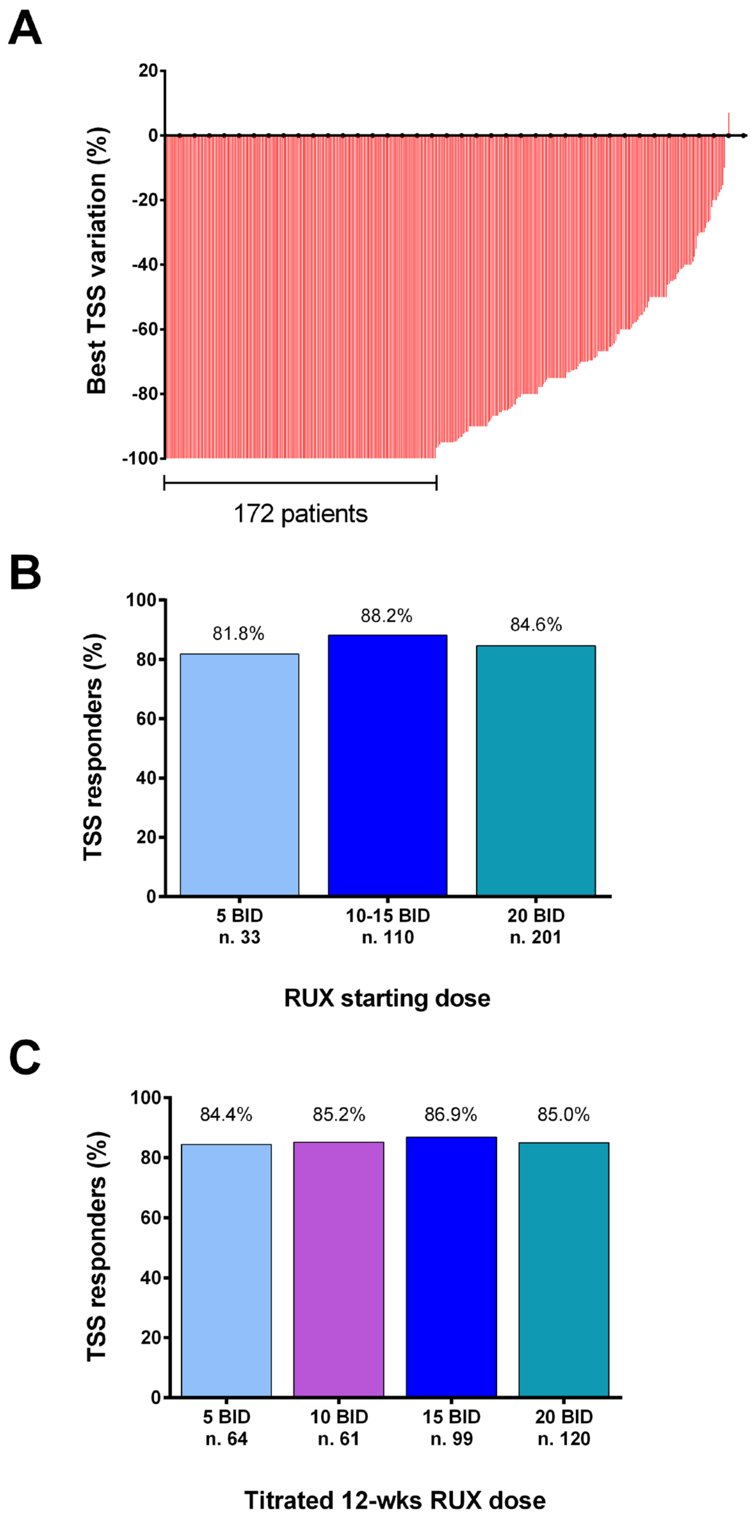

Supplementary Figure 2: Symptoms response. Best percent change from baseline in Total Symptom Score (TSS) at any time **(A)**. Each bar represents data from an individual patient. Percentage of patients achieving a symptoms response at 6 months according to ruxolitinib starting **(B)** and titrated **(C)** dose during the first 12 weeks of treatment.

Supplementary Table 1: Comparison of spleen and symptoms responses observed in prospective studies and in the present "real-life" study

Clinical trial (n. of patients)	Percentage of patients at intermediate-1 IPSS risk	Spleen response at week 24	Definition of spleen responses	Symptom response at week 24	Definition of symptoms responses
COMFORT-I (n. 155)	0	41.9%	≥35% volume reduction (MRI)	45.9%	2013 IWG-MRT criteria (≥50% TSS reduction from baseline)
COMFORT-II (n. 146)	0	32%	≥35% volume reduction (MRI)	N.R.	Improvement in different scales
JUMP (n. 1144)	14.2%	56.9%	≥50% length reduction*	44% - 52%	Minimally important difference according to different scales
ROBUST (n. 48)	29.2%	54.2%	≥50% length reduction*	54.2%	2013 IWG-MRT criteria (≥50% TSS reduction from baseline)
Present Study (n. 408)	15.7%	34.9%	2013 IWG-MRT criteria*	85.5%	2013 IWG-MRT criteria (≥50% TSS reduction from baseline)

*Spleen length was assessed by palpation. TSS: 10-item Total Symptom Score.
